# Association Between Nutritional Indices and Long-Term Outcomes in Patients Undergoing Isolated Coronary Artery Bypass Grafting

**DOI:** 10.7759/cureus.16567

**Published:** 2021-07-22

**Authors:** Omer Tasbulak, Arda Guler, Mustafa Duran, Anil Sahin, Umit Bulut, Yalcin Avci, Ali R Demir, Serkan Kahraman, Unal Aydin, Mehmet Ertürk

**Affiliations:** 1 Cardiology, University of Health Sciences, Mehmet Akif Ersoy Thoracic and Cardiovascular Surgery Training and Research Hospital, Istanbul, TUR; 2 Cardiology, University of Health Sciences, Konya Training and Research Hospital, Konya, TUR; 3 Cardiovascular Surgery, University of Health Sciences, Mehmet Akif Ersoy Thoracic and Cardiovascular Surgery Training and Research Hospital, Istanbul, TUR

**Keywords:** : coronary artery bypass surgery, prognostic nutritional index (pni), geriatric nutritional risk index (gnri), controlling nutritional status (conut), cabg

## Abstract

Background

It is well known that approximately 20% of patients who undergo cardiac surgery experience weight loss in postoperative period. However, there is a lack of data on postoperative consequences of malnutrition. This study aimed to investigate the relationship between nutritional status and long-term outcomes in patients undergoing isolated coronary artery bypass grafting (CABG).

Material and methods

A total of 586 patients who underwent isolated CABG in our center between January 2015 and March 2016 were included in this study. The primary outcome was major adverse cardiac and cerebrovascular events (MACCE) defined as a composite of all-cause death, non-fatal myocardial infarction (MI), and stroke. Patients were divided into two groups based on their MACCE outcomes. Prognostic nutritional index (PNI), geriatric nutritional risk index (GNRI), and controlling nutritional status (CONUT) scores were used to show the nutritional status.

Results

The mean follow-up time of the whole study group was 38.08 ± 13.4 months. The follow-up time was 39 ± 13 months in patients with mortality, while it was 20 ± 15 months in those without mortality. The PNI and GNRI values were lower in patients with major adverse cardiac and cerebrovascular events (MACCE) compared to patients without MACCE. The median CONUT score was higher in patients with MACCE.

Conclusion

Our study showed that nutritional indices including PNI, CONUT, and GNRI were associated with long-term MACCE and mortality in patients who underwent isolated CABG. The use of these scores in order to predict prognosis in patients treated with CABG seems to be an applicable method in clinical practice.

## Introduction

Cardiovascular diseases are the leading cause of death and disability worldwide. Coronary artery disease (CAD) takes the lead in this group of diseases [1]. With regard to the treatment of CAD, coronary artery bypass grafting (CABG) emerged as a convenient treatment modality together with medical therapies and percutaneous coronary intervention (PCI). Despite the improvements in percutaneous techniques, CABG still remains the preferred method over PCI especially in diabetic patients with multiple vessels or left main coronary artery disease [2]. It is well known that approximately 20% of patients who undergo cardiac surgery experience weight loss in postoperative period. The stress response to surgery and other comorbid conditions are underlying factors that provoke weight loss and contribute to the state of malnutrition [3].

Malnutrition is a major public health problem and is estimated to affect approximately 30% to 70% of hospitalized patients. It has an adverse effect on the cardiovascular system, immunological system, endocrine system, gastrointestinal system, and on the healing process during the recovery period [4]. There are several nutritional indices that have been developed to determine the nutritional status of an individual. Among them, the controlling nutritional status (CONUT) score [2], geriatric nutritional risk index (GNRI) [3], and prognostic nutritional index (PNI) are the most useful markers which provide simple, effective, and objective assessments of nutritional status [4]. According to recent studies, these parameters have been shown to be associated with predictors of mortality and morbidity in patients with prior history of heart failure [5-7]. Studies also confirmed that these parameters were also associated with clinical outcomes in patients with CAD [7,8].

There are some publications investigating the effect of these malnutrition criteria on CAD, and especially on mortality and long-term outcomes after PCI [7-9]. Furthermore, they have been shown to be effective in predicting mortality in patients undergoing CABG. On the other hand, there is a paucity of data in terms of correlation between nutritional indices and long-term outcomes and major adverse cardiovascular events in patients undergoing isolated CABG. This study aimed to demonstrate the predictive value of these malnutrition criteria for adverse cardiovascular events in patients undergoing isolated CABG.

## Materials and methods

This work was a retrospective observational study that complied with the Declaration of Helsinki and was approved by the appropriate health authorities, independent ethics committees, and independent review boards (Mehmet Akif Ersoy Thoracic and Cardiovascular Surgery Training and Research Hospital Local Ethics Committee; date: 22/12/2020; decision no: 2020/83) in our center.

A total of 586 patients between the ages of 35 years and 80 years, who underwent isolated CABG in our center between January 2015 and March 2016, were included in this study. Patients were analyzed retrospectively. Exclusion criteria included emergency procedures, additional valve surgery, active infection(s), recent major surgical procedure or trauma, previously proved systemic inflammatory disease, malignancy, and presence of end-stage renal and/or liver failures.

Demographic data, clinical variables, and medication use were collected from our institutional database. In addition, comorbidities of the patients such as chronic obstructive pulmonary disease, peripheral artery disease, atrial fibrillation, diabetes mellitus, hypertension, and myocardial infarction were recorded. Blood samples were collected in the early morning after overnight fasting, and blood pressure (BP) was measured upon admission. Patients with blood pressure (BP; 140/90 mmHg) or those receiving antihypertensive drugs were regarded as hypertensive. Dyslipidemia was defined as low-density lipoprotein cholesterol (LDL-C) >140 mg/dl, high-density lipoprotein cholesterol (HDL-C) >40 mg/dl, triglycerides >150 mg/dl, or those receiving drugs for hyperlipidemia.

The primary outcome was major adverse cardiac and cerebrovascular events (MACCE) defined as a composite of all-cause death, non-fatal myocardial infarction (MI), and stroke. Secondary outcomes are also defined as hospitalization owing to heart failure and repeat revascularization. Clinical follow-up included a review of medical charts, telephone contact, and questionnaires sent to patients or their families. Mortality data were collected from the medical records of patients who died or who were treated at our institution, and details and causes of death were obtained from other hospitals to which patients had been admitted. Cardiac death was defined as death from CAD, cardiogenic shock, or sudden death. Non-cardiac death was defined as death from other causes. MI was defined as evidence of myocardial necrosis in a clinical setting consistent with myocardial ischemia. Patients were divided into two groups based on their MACCE outcomes.

Nutritional indices assessment

Objective nutritional index (geriatric nutritional risk index {GNRI}, prognostic nutritional index {PNI}, and controlling nutritional status {CONUT}) scores were cal­culated at the time of hospital admission. The GNRI was calculated using a previously reported method: GNRI = 14.89 x serum albumin (g/l) + 41.7 x body weight (kg)/ideal body weight (kg) [10]. Height and weight were measured within 24 hours before surgery. The ideal body weight was calculated as follows: body height - 100 - ({body height -150}/4) for males, and body height - 100 - ({body height - 150}/2.5) for females. The PNI was calculated using the formula: 10 x serum albumin (g/dL) + 0.005 x total lymphocytes (count per mm^3^) [11]. The CONUT score consists of three variables: serum albumin, total cholesterol, and total lymphocyte count. Patients with CONUT scores of 9-12, 5-8, and 2-4 were considered to have a severe, moderate, and mild risk of malnutrition, respectively [12]. Those with a score of 0-1 were considered to have nor­mal nutritional status. The patients were divided into two groups as those with normal CONUT scores and those with mild - moderate - severe CONUT scores. Body mass index (BMI) was calculated using the formula: BMI = weight (kg)/height (m)^2^.

Statistical analysis

Statistical analysis was performed using the IBM SPSS Statistics for Windows, Version 21.0 software (Armonk, NY: IBM Corp.). The data were expressed as n (%) for categorical variables. The Pearson chi-square and Fisher exact tests were performed for categorical variables. After normal distribution was analyzed with the Kolmogorov-Smirnov test, the data were expressed as median (25th and 75th percentiles) for variables without a normal distribution and mean ± SD for variables with normal distribution. The Student’s t-test was used for comparing quantitative variables with normal distribution, and the Mann-Whitney U test was used for comparing quantitative variables without normal distribution. Univariate and multivariate logistic regression analyses were used to determine the independent predictors of primary endpoints of the study (MACCEs). Receiver operating characteristic (ROC) curve analysis was conducted to determine the optimal PNI, GNRI, and CONUT values to indicate mortality and primary endpoints of the study (MACCEs) in terms of both sensitivity and specificity. The survival curve using the PNI, GNRI, and CONUT was analyzed using the Kaplan-Meier method, and statistical assessment was performed using the log-rank test. A p-value < 0.05 was considered statistically significant.

## Results

A total of 586 patients (mean age was 59.5; 78% of patients were male) with CAD and underwent isolated CABG were included. The mean follow-up time of the whole study group was 38.08 ± 13.4 months. The follow-up time was 39 ± 13 months in patients without mortality, while it was 20 ± 15 months in those with mortality. Additionally, the follow-up time was 38 ± 14 months in patients without MACCE, while it was 15 ± 9 months in those with MACCE. Baseline characteristics of the patients categorized by MACCE outcomes are listed in Table 1.

**Table 1:** Demographic, clinical, and nutritional characteristics of the patients EF: ejection fraction, CONUT: controlling nutritional status, MACCE: major adverse cardiac and cerebrovascular events

	MACCE (-) (n=533)	MACCE (+) (n=53)	p-Value
Age	59.5 ± 9.2	60.5 ± 8.0	0.474
Gender (women), n (%)	117 (22)	13 (24.5)	0.667
Chronic obstructive pulmonary disease, n (%)	73 (13.7)	8 (15.1)	0.778
Peripheral artery disease, n (%)	46 (8.6)	10 (18.9)	0.016
Atrial fibrillation, n (%)	11 (2.1)	1 (1.9)	0.703
Smoking, n (%)	175 (32.8)	26 (49.1)	0.018
Diabetes mellitus, n (%)	288 (54)	34 (64.2)	0.158
Hypertension, n (%)	411 (77.1)	44 (83)	0.325
Previous myocardial infarction, n (%)	90 (16.9)	9 (17)	0.986
Heart failure with reduced EF, n (%)	38 (7.1)	7 (13.2)	0.099
EF, (%)	60 (50-60)	55 (45-60)	0.060
Stroke, n (%)	42 (7.9)	1 (1.9)	0.081
Clinical admission, n (%)			
Acute coronary syndrome, n (%)	207 (38.8)	26 (49.1)	0.147
Stable chronic coronary syndrome, n (%)	326 (61.2)	27 (50.9)	
Prognostic nutritional risk index	55.45 (52.25 - 59.4)	43.35 (39.75 - 48.8)	<0.001
Geriatric nutritional risk index	58.64 (53.85 - 64.13)	56.06 (50.65 - 62.09)	0.019
CONUT	1 (0-2)	5 (4-6)	<0.001
CONUT group, n (%)			
Normal nutrition	391 (73.4)	5 (9.4)	<0.001
Mild malnutrition	136 (25.5)	15 (28.3)	
Moderate malnutrition	6 (1.1)	32 (60.4)	
Severe malnutrition	0 (0)	1 (1.9)	

Clinical history of the patients was similar in terms of hypertension, diabetes mellitus, previous MI, chronic kidney disease, peripheral arterial disease (PAD), heart failure, chronic lung disease, and stroke. Clinical history was also similar in terms of acute coronary syndrome or stable CAD. Patients with smoking and peripheral arterial disease were higher in the MACCE + group. PNI (MACCE {-}: 55.45 {52.25-59.4} vs. MACCE {+}: 43.35 {39.75-48.8}, p<0.001) and GNRI (MACCE {-}: 58.64 {53.85-64.13} vs. MACCE {+}: 56.06 {50.65-62.09}, p=0.019) values were significantly lower in patients with MACCE compared to patients without MACCE. The median CONUT score was higher in patients with MACCE (1 {0-2} vs. 5 {4-6}, p<0.001). The number of patients with normal CONUT score (391 {73.4%} vs. 5 {9.4%}, p<0.001) was lower in patients with MACCE. Table 2 provided laboratory findings of the patients.

**Table 2:** Laboratory findings of the patients MACCE: major adverse cardiac and cerebrovascular events, AST: serum aspartate aminotransferase, ALT: alanine aminotransferase

	MACCE - (n=533)	MACCE + (n=53)	p-Value
Glucose, mg/dl	114 (96-153)	120 (101-198)	0.094
Creatinine, mg/dl	0.9 (0.77-1.05)	0.9 (0.79-1.1)	0.433
Total cholesterol, mg/dl	180 (143-215)	181 (143-224)	0.692
Low-density lipoprotein, mg/dl	105 (76-140)	104 (71-143)	0.957
High-density lipoprotein, mg/dl	40 (34-47)	40 (35-49)	0.545
Triglyceride, mg/dl	147 (106-209)	156.5 (103-232)	0.765
Hemoglobin, g/dl	13.9 (12.6-15)	13.6 (12.1-14.3)	0.137
Platelet, 10^3^/mm^3^	258 (212-313)	251 (205-273)	0.217
White blood count, 10^3^/μl	8.49 (7.25-10.1)	8.14 (7.15-9.68)	0.558
Body mass index, kg/m^2^	27.85 (25.47-31.08)	27.38 (24.96-30.12)	0.264
AST, μ/l	17 (14-23)	17 (14-21)	0.369
ALT, μ/l	18 (14-25)	18 (14-21)	0.358
Albumin, mg/dl	4.4 (4.1-4.64)	3.2 (2.8-3.74)	<0.001
Neutrophil, k/μl	5.38 (4.34-6.59)	5.03 (4.37-6.38)	0.561
Lymphocyte, %	2.32 (1.88-2.9)	2.33 (1.98-2.69)	0.699

In laboratory parameters, the groups were similar with respect to glucose, creatinine, cholesterol counts, liver enzymes, hemoglobin, and platelet counts. BMI was similar between the groups. Additionally, the nutritional statuses of patients with secondary outcomes were demonstrated in Table 3.

**Table 3:** Nutritional status of the patients when compared to secondary outcomes PNI: prognostic nutritional index, CONUT: controlling nutritional status, GNRI: geriatric nutritional index

	Secondary outcomes (-) (n=544)	Secondary outcomes (+) (n=42)	p-Value
PNI	55.4 (51.83-59.33)	47.1 (43.2-51.65)	<0.001
CONUT	1 (0-2)	3 (2-5)	<0.001
CONUT group, n (%)			
Normal nutrition	390 (71.7)	6 (14.3)	<0.001
Mild malnutrition	128 (23.5)	23 (54.8)	
Moderate malnutrition	25 (4.6)	13 (31.0)	
Severe malnutrition	1 (0.2)	0 (0)	
GNRI	58.47 (53.81-64.12)	54.83 (52.3-59.92)	0.039

The PNI (55.4 {51.83-59.33} vs. 47.1 {43.2-51.65}, p<0.001) and GNRI (58.47 {53.81-64.12} vs. 54.83 {52.3-59.92}, p=0.039) values were lower in patients with secondary outcomes compared to patients without secondary outcomes. The median CONUT score was higher in patients with secondary outcomes (1 {0-2} vs. 3 {2-5}, p<0.001). The number of patients with normal CONUT score (390 {71.7%} vs. 6 {14.3%}, p<0.001) was lower in patients with secondary outcomes.

**Figure 1:**
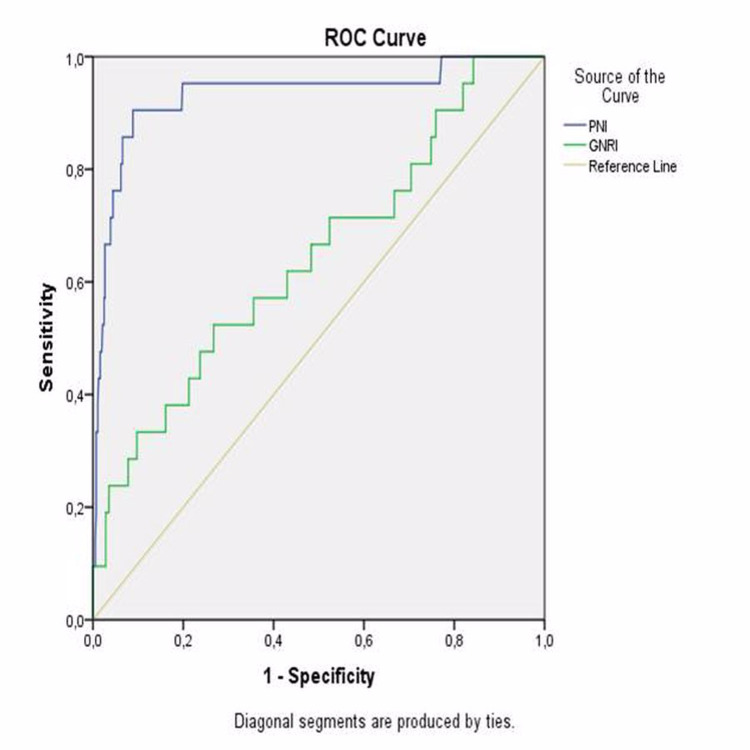
ROC curves of PNI and GNRI as a predictor of mortality ROC: receiver operating characteristic, PNI: prognostic nutritional index, GNRI: geriatric nutritional risk index

ROC curve analyses were conducted to determine the optimal PNI, GNRI, and CONUT cut-off value to indicate mortality and MACCE separately. The highest combined sensitivity and specificity value crossed the curve at 46,97 (sensitivity: 90.5%; specificity: 91.2%) for PNI to indicate mortality (Figure 1).

**Figure 2:**
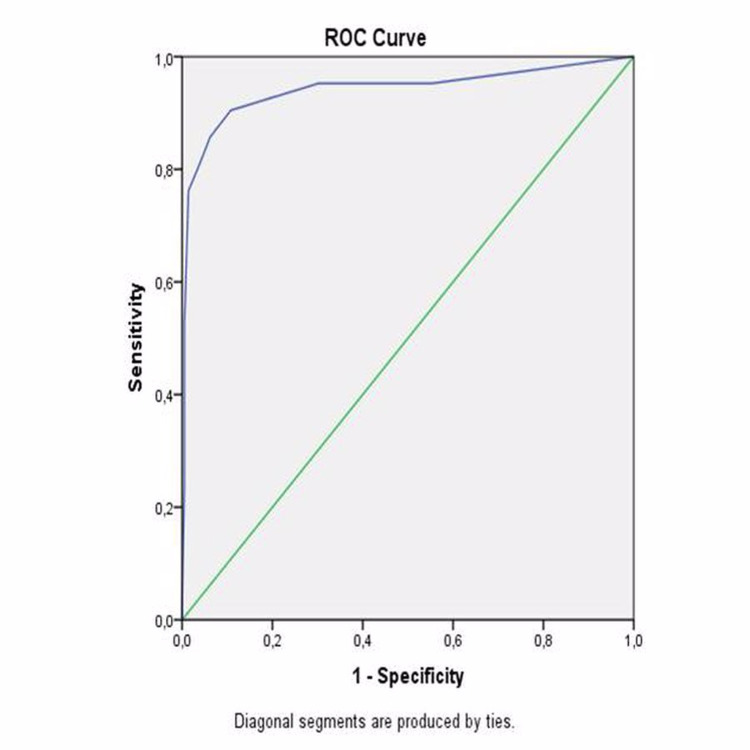
ROC curve of CONUT score as a predictor of mortality ROC: receiver operating characteristic, CONUT: controlling nutritional status

The area under the curve was 0.932 (95% confidence interval {CI}: 0.861-1.000; p<0.001). The highest combined sensitivity and specificity values crossed the curve at 54.06 (sensitivity: 52.4%; specificity: 73.3%) for GNRI to indicate mortality (Figure 1). The area under the curve was 0.644 (95% CI: 0.516-0.772; p=0.025). The highest combined sensitivity and specificity values crossed the curve at 2.5 (sensitivity: 90.5%; specificity 89.2%) for CONUT to indicate mortality (Figure 2).

**Figure 3:**
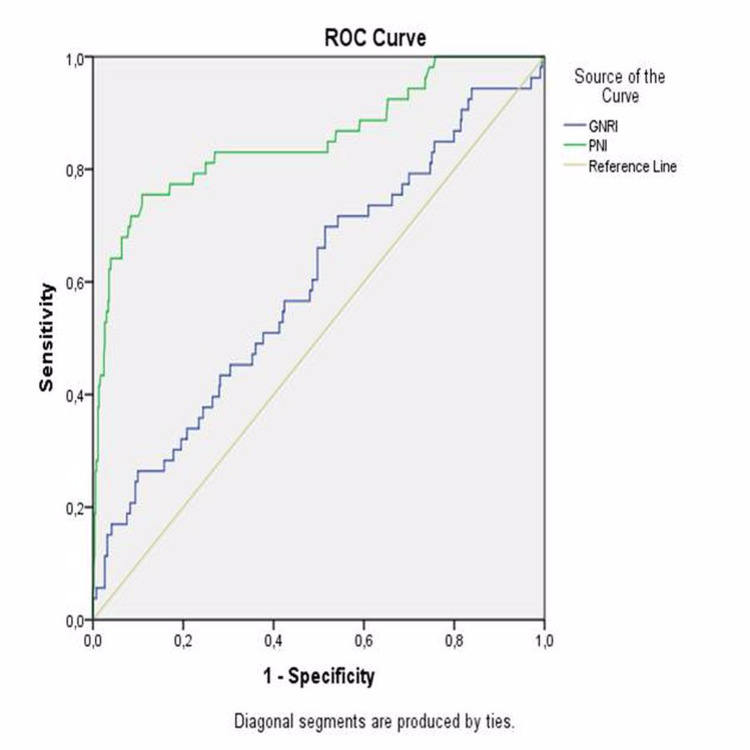
ROC curves of PNI and GNRI as a predictor of MACCE ROC: receiver operating characteristic, PNI: prognostic nutritional index, GNRI: geriatric nutritional risk index, MACCE: major adverse cardiac and cerebrovascular events

The area under the curve was 0.941 (95% CI: 0.868-1.000; p<0.001). The highest combined sensitivity and specificity values crossed the curve at 48.8 (sensitivity: 75.5%; specificity: 89.1%) for PNI to indicate MACCE (Figure 3). The area under the curve was 0.85 (95% CI: 0.788-0.919; p<0.001). The highest combined sensitivity and specificity values crossed the curve at 59.01 (sensitivity: 69.8%; specificity: 48.6%) for GNRI to indicate MACCE (Figure 3).

**Figure 4:**
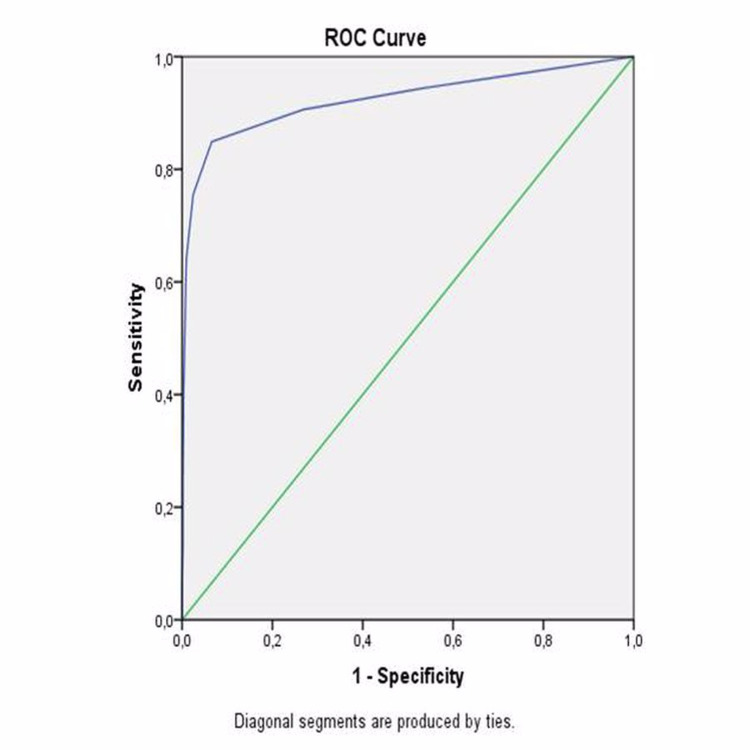
ROC curve of CONUT score as a predictor of MACCE ROC: receiver operating characteristic, CONUT: controlling nutritional status, MACCE: major adverse cardiac and cerebrovascular events

The area under the curve was 0.598 (95% CI: 0.514 - 0.681; p=0.019). The highest combined sensitivity and specificity values crossed the curve at 2.5 (sensitivity: 84.9%, specificity: 93.4%) for CONUT to indicate MACCE (Figure 4).

**Figure 5:**
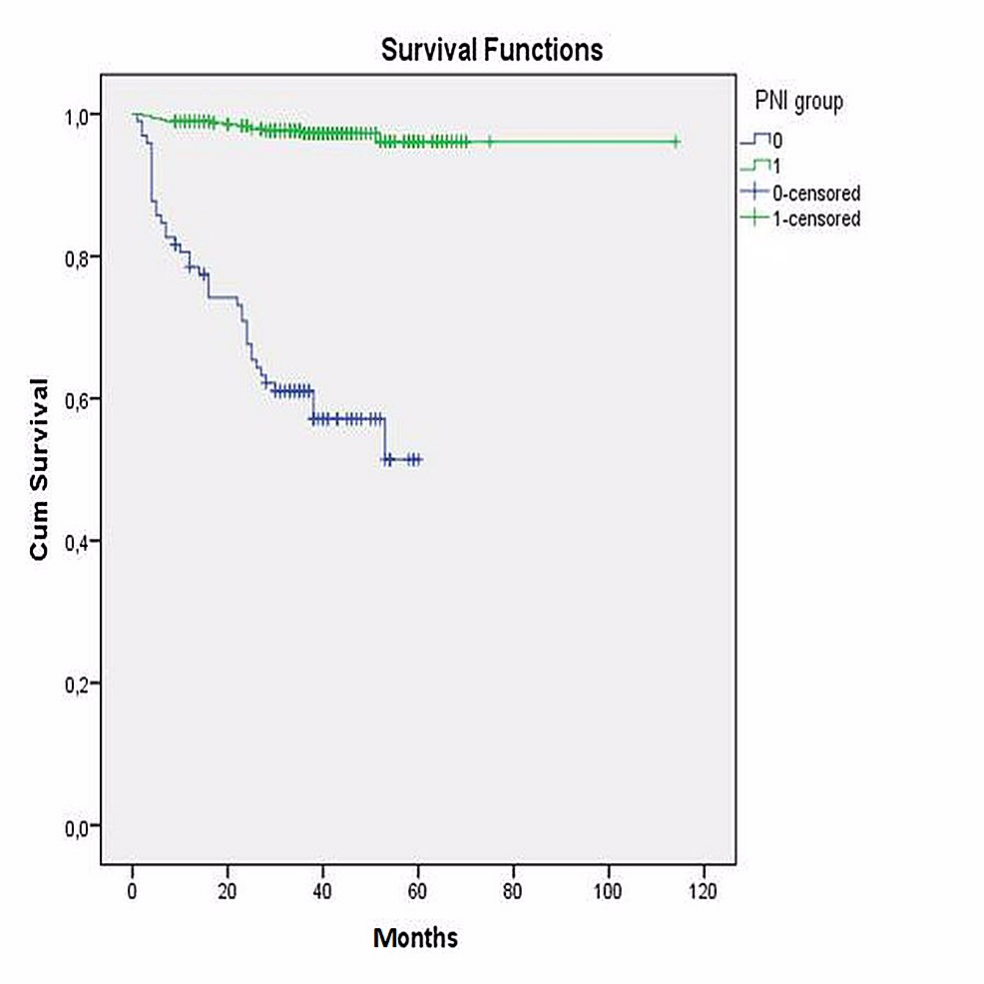
Kaplan-Meier analyses of PNI PNI: prognostic nutritional index

**Figure 6:**
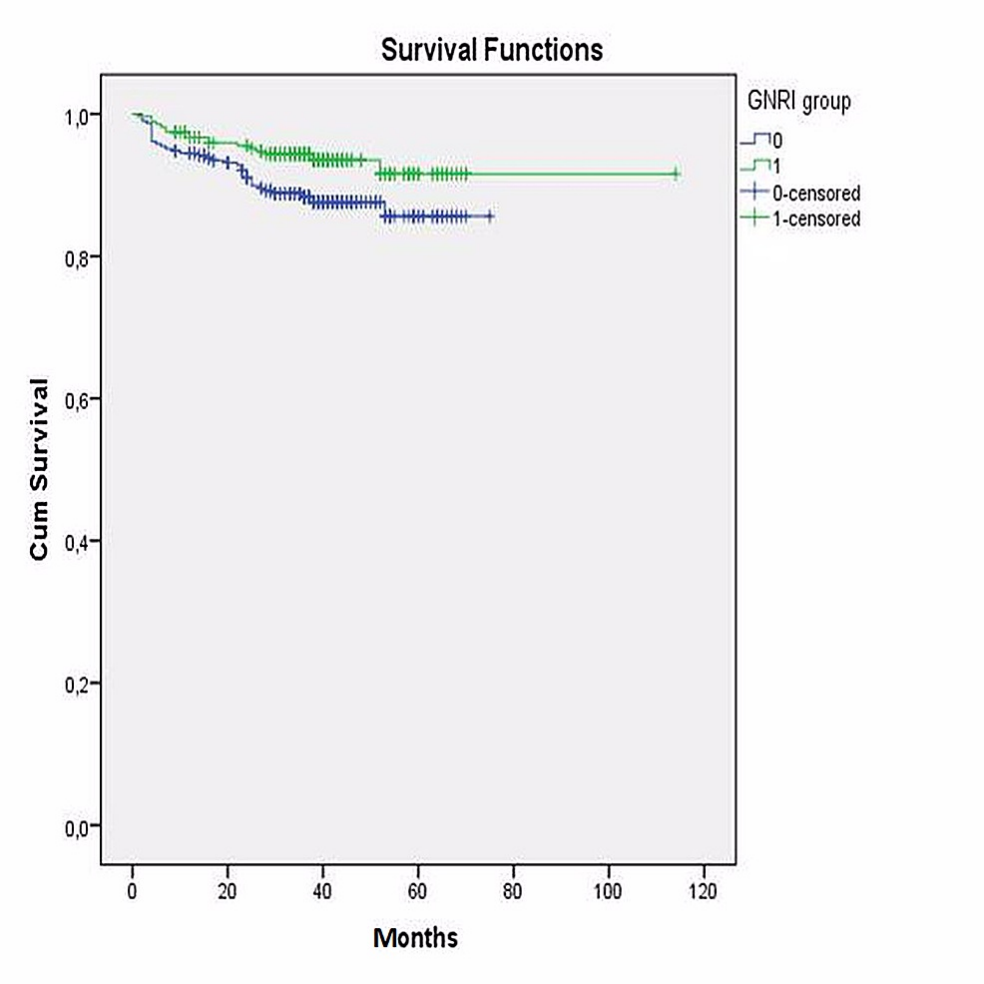
Kaplan-Meier analyses of GNRI GNRI: geriatric nutritional risk index

The area under the curve was 0.924 (95% CI: 0.872 - 0.976; p<0.001). Then, the whole study group was divided into two groups according to their PNI and GNRI cut-off values separately. Kaplan-Meier survival analysis also revealed that long-term survival was found to be significantly decreased in patients with a lower PNI (log-rank: p<0.001) (Figure 5) and a lower GNRI (log-rank: p=0.032) (Figure 6).

**Figure 7:**
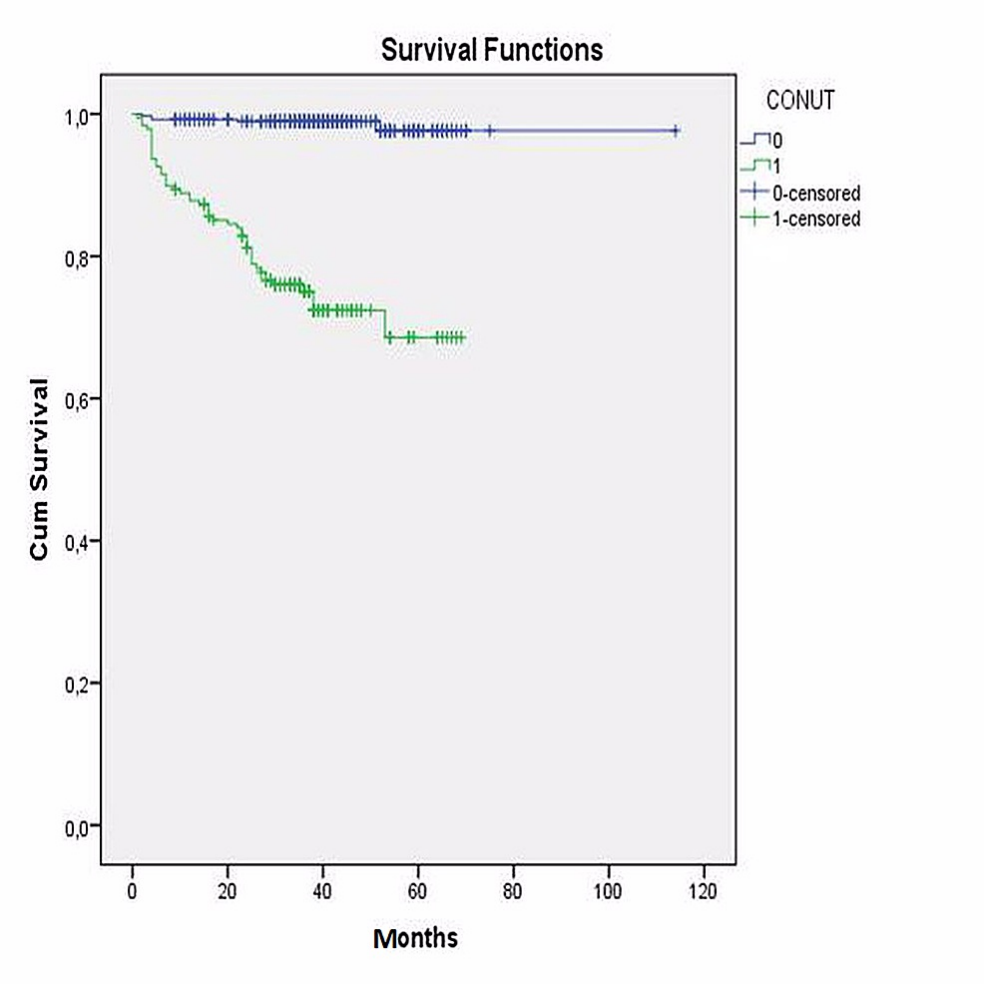
Kaplan-Meier analyses of CONUT score CONUT: controlling nutritional status

Additionally, the entire study group was divided into two groups according to the CONUT value, as group 1 with normal CONUT score and group 2 with higher CONUT score indicating different level risk of malnutrition. Kaplan-Meier survival analysis also revealed that long-term survival was found significantly decreased in patients with a higher CONUT score (log-rank: p<0.001) (Figure 7).

Finally, independent predictors of MACCE were analyzed with logistic regression analyses. After logistic regression analyses, significant variables found in the univariate analysis were entered into multiple logistic regression. Two separate models were conducted for PNI and GNRI separately. The multivariate logistic regression analysis indicated that smoking (OR: 2.087; 95% CI: 1.040-4.188; p=0.038), PNI (OR: 0.774; 95% CI: 0.730-0.819; p<0.001), and PAD (OR: 3.253; 95% CI: 1.250-8.462; p=0.016) were found to be independent predictors of MACCE (Table 4).

**Table 4:** Multivariate analyses of parameters in predicting MACCE MACCE: major adverse cardiac and cerebrovascular events

	Odds ratio	95% confidence interval	p-Value
Smoking status	2.087	1.040-4.188	0.038
Peripheral arterial disease	3.253	1.250-8.462	0.016
Prognostic nutritional index	0.774	0.730-0.819	<0.001

Additionally, smoking (OR: 3.557; 95 CI: 1.417-8.927; p=0.007), CONUT score (OR: 3.587; 95% CI: 2.707-4.752; p<0.001), and PAD (OR: 4.627 95% CI: 1.424-15.032; p=0.011) were found to be independent predictors of MACCE (Table 5).

**Table 5:** Multivariate analyses of parameters in predicting MACCE CONUT: controlling nutritional status, MACCE: major adverse cardiac and cerebrovascular events

	Odds ratio	95% confidence interval	p-Value
Smoking status	3.557	1.417-8.927	0.007
Peripheral arterial disease	4.627	1.424-15.032	0.011
CONUT score	3.587	2.707-4.752	<0.001

## Discussion

In this study, the relationship between the objective nutritional indices and long-term MACCE in patients undergoing isolated CABG was investigated. Based on our results, we found a strong association between nutritional status and long-term MACCE and mortality rates in patients who underwent isolated CABG. Our study demonstrated that PNI, CONUT, and GNRI scores were significant prognostic nutritional markers for patients undergoing isolated CABG. This indicates that these nutritional parameters could be used as an easy and practical indicator for identifying long-term outcomes in this patient population.

With respect to the treatment of CAD, medical treatment, PCI, and open surgery are widely accepted methods, which have been valid for years [13,14]. Some studies have shown that CABG was a much more effective treatment modality in some patient groups [15,16]. Some of the biggest difficulties related to CABG are intraoperative and postoperative complications along with the development of adverse cardiovascular events. Besides, in some patients, valve surgery or additional cardiac procedures are needed who undergo CABG. Not only procedure-related risks, but the frequency of postoperative complications in these patient populations were higher than those who underwent isolated CABG [17]. Therefore, only patients who underwent isolated CABG were evaluated in our research in order to avoid long-term complications related to additional surgical procedures.

Nutritional status has a crucial role in the continuation of vital tissue and organ functions in the body. In malnutrition, which is defined as the deterioration of nutritional status, a decrease in the food intake required for the maintenance of basic organ functions, and a deterioration in the protein cycle observed [4]. Malnutrition has been reported to be associated with poor prognosis, especially in elderly patients, patients with malignancy, and patients with chronic diseases such as ischemic stroke, heart failure, or coronary artery disease [18]. This has led to the pursuit of various proposed markers in order to evaluate nutritional status. Some of these are known as PNI, CONUT, and GNRI scores. The relationship between those nutritional parameters and the development of cardiovascular diseases has been demonstrated by various studies so far [7,9,19,20].

PNI, which is one of the important objective nutritional screening tools, has been used as a predictive marker for nutritional status and surgical risk in various patient populations including patients who underwent gastrointestinal surgery [11]. PNI is a marker based on the assessment of serum albumin levels and the lymphocyte count. It was shown that lower PNI levels were associated with postoperative mortality in patients undergoing CABG [11]. According to a study conducted by de Ulíbarri et al., lower PNI values ​​were associated with higher ACE and mortality rates in patients with prior history of heart failure [12]. Outcomes of our study were compatible with the results of previous studies, which revealed lower PNI scores were associated with higher rates of MACCE in patients who underwent isolated CABG.

Another objective nutritional assessment tool is the CONUT score. This score is derived from the values ​​of serum albumin, total cholesterol, and lymphocyte counts [12]. Albumin is an inflammation marker that plays an important role in endothelial function and lipoprotein structure. Low albumin levels may cause atherosclerotic disease through endothelial dysfunction [18]. This situation may worsen pre-existing CAD and increase the risk of future adverse cardiovascular events. The relationship between high cholesterol levels, especially elevated LDL cholesterol levels, and atherosclerotic heart disease has been known for years [19]. However, it has been reported that low LDL values ​​in some patients also increase vascular inflammation and in some cases, this may even be an indicator of advanced atherosclerosis [20]. Malnutrition can also provoke this condition by creating low level of cholesterol concentrations [13]. Another parameter of this score is the lymphocytic count. Low lymphocyte count is an indicator of neurohormonal dysfunction in the body and is associated with low immunity in patients with CAD. This may contribute to the development of atherosclerosis by increasing vascular stress [14]. It may also be associated with poor prognosis in patients with CAD. The CONUT score was first defined by Bouillanne et al. in 2005 and found to be an important marker in terms of showing the nutritional status of hospitalized patients [10]. Studies conducted in the following years have revealed that the CONUT score can also be a useful parameter indicative of the clinical condition for patients with cardiovascular disease. CONUT score determined both as a factor increasing in-hospital mortality in patients with acute heart failure and chronic failure, and as a predictor of long-term mortality [12,15]. In a study by Keskin et al., CONUT score was independently associated with an increased future risk of acute myocardial infarction, cardiovascular death, heart failure, major advance cardiovascular events, and total cardiovascular events, extending the predictive value of nutrition scoring in CAD patients after PCI [5]. According to our study, MACCE and mortality were observed less frequently in patients with low CONUT scores. In addition, high CONUT score was found to be associated with worse prognosis in long-term follow-up, and lower CONUT score was associated with higher survival in patients who underwent isolated CABG.

GNRI is a nutritional evaluation method determined by objective criteria that predicts mortality and morbidity in elderly patients and patients with chronic diseases. Bouillanne et al. first described this parameter in 2005. The parameter was used as a predictive marker for the effects of complications secondary to malnutrition on in-hospital mortality at that time [10]. Apart from elderly patients, it has been shown to be an effective marker for showing nutritional status in patients less than 65 years of age. There were also studies showing that GNRI was associated with mortality in patients with prior history of heart failure [19]. According to our data, MACCE was observed more frequently in patients with isolated CABG and low GNRI scores, and lower survival was found in the long-term follow-up of these patients.

In our study, three different nutritional assessment tools were used. Gürbak et al. previously researched the outcome of all three tools on patients who underwent aortic valve replacement. Their study demonstrated the association between PNI, GNRI, CONUT score, and outcome after aortic valve replacement [21]. Similar to their study, we have also demonstrated the association of nutritional status with three different tools and cardiovascular events after isolated CABG surgery.

Limitations

There are some limitations to our study. First and foremost, the retrospective nature of the study inadvertently limited the diagnostic value of the research. Second, some factors such as intraoperative parameters that may affect the prognosis in patients undergoing CABG were not evaluated in this study. Third, some hormonal parameters may affect nutritional status, and in our study, especially serum catecholamine and steroid hormone levels were not evaluated. Due to the fact that it is a retrospective study, especially the optimal medical treatment could not be followed, this may have affected the results as a factor affecting the long-term outcomes. Furthermore, the nutritional status and malnutrition scores of the patients may change in the long-term follow-up. However, in our study, the scores calculated during the hospitalization and operation periods were considered. Because of these limitations, prospectively designed and larger studies are mandatory in order to verify these results.

## Conclusions

Our study showed that nutritional indices including PNI, CONUT, and GNRI are associated with long-term MACCE and mortality in patients undergoing isolated CABG. The use of these scores in order to predict prognosis in patients treated with CABG seems to be an applicable method in clinical practice. It is important to evaluate nutritional parameters in the preoperative period in patients who will undergo isolated CABG. In this period, optimizing the nutritional status and performing surgery with the appropriate nutritional status of the patients may help to reduce postoperative and long-term outcomes. However, depending on the limitations of the study, multi-center and prospective studies are needed to validate our findings.
